# Supplementary material for: Fluorescence In Situ Hybridization in Primary Diagnosis of Biliary Strictures: A Single-Center Prospective Interventional Study
Source: Biomedicines. 2023 Mar 2;11(3):755. doi: 10.3390/biomedicines11030755 (PMC10045065; doi:10.3390/biomedicines11030755)
Supplement: Supplementary file 1 [file biomedicines-11-00755-s001.zip › biomedicines-2215381-supplementary.pdf]

Supplementary Table S1: *Statistical performances of brush cytology (BC) vs. BC + FISH (malignant diagnosis group)*

	**Malignant outcome**								
n= 74	Pancreatic tumors*			Cholangiocarcinoma			Others**		
	BC	BC + FISH	p	BC	BC + FISH	p	BC	BC +FISH	p
Sensitivity	0.316	0.421	0.343	0.458	0.680	0.117	0.300	0.400	0.639
(95% CI)	(0,175-0,486)	(0.263-0.592)		(0.256-0.672	(0.465-0.851)		(0.067-0.653)	(0.122-0.738)	
Specificity	0.852	0.741	0.311	0.852	0.741	0.311	0.852	0.741	0.311
(95% CI)	(0.663-0.958)	(0.537-0,889)		(0.663-0.958)	(0.537-0.889)		(0.663-0.958)	(0.537-0.889)	
PPV	0.750	0.696	0.712	0.733	0.708	0.866	0.429	0.364	0.783
(95% CI)	(0.476-0.927)	(0.471-0.868)		(0.449-0.922)	(0.489-0.874)		(0.099-0.816)	(0.109-0.692)	
NPV	0.469	0.476	0.940	0.639	0.714	0.535	0.767	0.769	0.986
(95% CI)	(0.325-0.617)	(0.320-0.636)		(0.462-0.792)	(0.513-0.868)		(0.577-0.901)	(0.563-0.910)	
Accuracy	0.538	0.554	0.855	0.667	0.721	0.622	0.703	0.649	0.631
(95% CI)	(0.410-0.663)	(0.725-0.677)		(0.521-0.792)	(0.569-0.829)		(0.530-0.841)	(0.475-0.798)	

FISH = fluorescence in situ hybridization, BC = brush cytology, PPV = positive predictive value, NPV = negative predictive value, CI = confidence interval, p = p-value (BC vs. BC + FISH),

**Pancreatic tumors = pancreatic ductal adenocarcinoma (89.5%), malignant pancreatic cystic tumors (7.9%) and pancreatic lymphoma (2.6%).*

***Others = B cells malignant / Lymphoma (30%), gallbladder adenocarcinoma (20%), ampullary adenocarcinoma (20%), metastatic disease (liver or malignant lymph nodes compression) (30%).*

__Supplementary Table S2__: Statistical performances of brush cytology and brush cytology added to each chromosome in overall study population (I) vs. population with intrinsic strictures (II)

	Sensitivity (95% CI)		***Specificity (95% CI)***		***Accuracy (95% CI)***		***PPV***		***NPV***		***AUC***	
	I	***II***	***I***	***II***	***I***	***II***	***I***	***II***	***I***	***II***	***I***	***II***
BC	0.361	0.480	0.852	0.867	0.495	0.625	0.867	0.857	0.333	0.500	0.606	0.673
	(0.251-0.483)	(0.278-0.687)	(0.663-0.958	(0.595-0.983)	(0.393-0.597	(0.458-0.773)						
BC + Chr 3	0.575	0.808	0.741	0.733	0.620	0.780	0.857	0.840	0.392	0.688	0.658	0.771
	(0.454-0.690)	(0.606-0.934)	(0.537-0.889)	(0.449-0.922)	(0.517-0.715)	0.624-0.894						
BC + Chr 7	0.658	0.769	0.571	0.667	0.634	0.732	0.800	0.800	0.390	0.625	0.614	0.718
	(0.537-0.765)	(0.563-0.910)	(0.372-0.755)	(0.384-0.882)	(0.532-0.727)	(0.571-0.858)						
BC + Chr 17	0.389	0.480	0.852	0.867	0.515	0.625	0.875	0.857	0.343	0.500	0.620	0.673
	(0.276-0.511)	(0.278-0.687)	(0.663-0.958)	(0.595-0.983)	(0.412-0.617)	(0.458-0.773)						
BC + p21	0.417	0.600	0.852	0.867	0.535	0.700	0.882	0.882	0.354	0.565	0.634	0.733
	(0.302-0.339)	(0.387-0.789)	(0.663-0.958)	(0.595-0.983)	(0.432-0.636)	(0.535-0.834)						

I = overall study population (n = 102); II = population with intrinsic strictures (n = 40); PPP = positive predictive value; NPV = negative predictive value; AUC = area under the curve; CI = confidence interval; BC = brush cytology; Chr 3, 7, and 17 = chromosomes 3, 7, and 17; p21 = chromosomal locus 9p21

Supplementary table S3: Demography of the patients died during the study Follow up.

NP	Final diagnosis	Gender	Survival* (Months)	Age (year)	Stenosis
1	Cholangiocarcinoma in PSC	Male	6	50	Proximal
2	Pancreatic head carcinoma	Male	3	69	Distal
3	Cholangiocarcinoma	Female	5	66	Distal
4	Cholangiocarcinoma	Male	5	76	Distal
5	Pancreatobiliary carcinoma	Female	3	68	Distal
6	Cholangiocarcinoma in PSC	Female	11	44	Proximal
7	Pancreatic head carcinoma	Male	2	78	Distal
8	Cholangiocarcinoma	Male	9	72	Proximal
9	Gallbladder carcinoma	Male	6	71	Distal
10	Lung Carcinoma/ CP**	Male	6	73	Distal***
11	Rectal Carcinoma (liver metastases, lymphadenopathy)	Female	3	59	Distal

*NP=Patient numbers in chronological order of death, * survival counted after initial tissue sampling, PSC= Primary sclerosing cholangitis, ** He died for lung cancer and not because of stenosis due to Chronic pancreatitis (CP), *** non- malignant stenosis*

Supplementary Table S4: *Comparison of those Papanicolaou categories considered as positive for malignancy for purposes of this study and the study's final clinical diagnosis after conclusive 12-month follow-up and/or postoperative histology*

Final clinical diagnosis	*PAP V*	*PAP VI*
Malignant (%)	83	100
Benign (%)	17	0

PAP (V, VI) = Categories of Papanicolaou Society's nomenclature for pancreatobiliary cytology, Malignant = malignant definitive clinical diagnosis, Benign = benign definitive clinical diagnosis

Supplementary Table S5: *Statistical performances of forceps biopsy and brush cytology plus forceps biopsy*

	Biopsy (n = 44)	*BC*	*BC + Biopsy*	*P*(BC vs. BC + Biopsy)*
Sensitivity (95% CI)	0.406 (0.237-0.594)	0.361 (0.251-0.483)	0.667 (0.504-0.804)	0.002
Specificity (95% CI)	0.917 (0.615-0.998)	0.852 (0.663-0.958)	0.733 (0.449-0.922)	0.347
PPV	0.929	0.867	0.875	1.000
NPV	0.367	0.333	0.440	0.342
Accuracy (95% CI)	0.545 (0.388-0.696)	0.495 (0.393-0.597)	0.684 (0.548-0.801)	0.022

PPV = positive predictive value, NPV = negative predictive value, CI = confidence interval, biopsy = transpapillary fluoroscopy guided forceps biopsy, BC = brush cytology, P= p-value (BC vs. BC + Biopsy)*

Supplementary table S6 -*Statistical performances of brush cytology (BC) vs. FISH vs. Biopsy vs. different combinations (study population)*

	BC	FISH	Biopsy	BC+ Biopsy	BC + FISH + Biopsy
Sensitivity (95%CI)	0.361 (0.251-0.483)	0.311 (0.208-0.429)	0.406 (0.237-0.594)	0.667 (0.504-0.804)	0.739 (0.589-0.857)

Specificity (95%CI)	0.852 (0.663-0.958)	0.821 (0.631-0.939)	0.917 (0.615-0.998)	0,733 (0.449-0.922)	0.588 (0.329-0.816)
PPV	0.867	0.821	0.929	0.875	0.829
NPV	0.333	0.311	0.367	0.440	0.455
Accuracy (95%CI)	0.495 (0.393-0.597)	0.451 (0.352-0.553)	0.545 (0.388-0.696)	0.684 (0.548-0.801)	0.698 (0.507-0.808)

PPV= positive predictive value, NPV = negative predictive value, CI = confidence interval, p = p-value, BC = brush cytology, FISH = fluorescence in situ hybridization.
